# Stereotactic body radiotherapy (SBRT) for high-risk central pulmonary metastases

**DOI:** 10.1186/s13014-016-0608-8

**Published:** 2016-02-27

**Authors:** Jonathan W. Lischalk, Ryan M. Malik, Sean P. Collins, Brian T. Collins, Ismael A. Matus, Eric D. Anderson

**Affiliations:** Department of Radiation Medicine, Lombardi Comprehensive Cancer Center, Georgetown University Hospital, Lower Level Bles, 3800 Reservoir Road, NW, Washington, DC 20007 USA; Division of Pulmonary, Critical Care, and Sleep Medicine, Pasquerilla Healthcare Center, Georgetown University Hospital, 5th floor, 3800 Reservoir Road, NW, Washington, DC 20007 USA

**Keywords:** Lung neoplasms, Metastasis, Primary bronchus, Stereotactic body radiotherapy, Pulmonary atelectasis

## Abstract

**Background and purpose:**

Radiotherapy of central lung tumors carries a higher risk of treatment-related toxicity and local failure. In the era of aggressive oligometastic management the exploration of the proper dose-fractionation for metastatic central lung tumors is essential.

**Materials and methods:**

Patients diagnosed with high-risk metastatic lesions of the central pulmonary tree comprised this single-institutional retrospective analysis. “High-risk” central pulmonary lesions were defined as those with abutment and/or invasion of the mainstem bronchus. All patients were treated using the CyberKnife SBRT system in 5 fractions to a total dose of 35 or 40 Gy.

**Results:**

Twenty patients were treated from 2008 to 2011 at Georgetown University Hospital. At a median follow up of 19 months, 1-year Kaplan-Meier local control and overall survival was 70 and 75 %, respectively. Late grade 2 or higher atelectasis was the most common treatment-related toxicity and was significantly associated with maximum dose to the mainstem bronchus. Gross endobronchial involvement was associated with significantly lower overall survival.

**Conclusions:**

Five-fraction SBRT to a total dose of 35 or 40 Gy appears to be a safe and effective management strategy for high-risk central pulmonary metastatic lesions, though care should be taken to limit the maximum point dose to the mainstem bronchus.

## Introduction

Management of symptomatic and/or compromising pulmonary metastases is a common clinical scenario encountered by oncologists. Although first-line treatment of metastatic disease is often systemic therapy there is a modern movement to more aggressively manage oligometastatic disease with local therapy [1, 2]. Long-term survival in carefully selected metastatic patients was initially established by the International Registry of Lung Metastases trial where surgical metastasectomy achieved a 26 % 10-year overall survival rate [3]. Central lung metastases are a particularly dangerous variant of pulmonary metastases and not only limit a patient’s life expectancy but can also profoundly impact their quality of life [4]. Unfortunately, treatment of these central lung tumors with high dose radiation carries an increased risk of treatment-related toxicity relative to more peripheral lung lesions [5, 6].

The elevated risk of toxicity with SBRT to central primary lung tumors has been previously demonstrated in the analysis of the prospective phase II trial for early stage inoperable non-small cell lung cancer (NSCLC) published by Timmerman et al. [5]. The reported toxicities included decline in PFTs, pneumonia, and pleural/pericardial effusions. Such toxicities are reflective of the high doses delivered to the proximal bronchial tree causing secondary atelectasis and nearby radiosensitive structures such as the esophagus, heart, and spinal cord [5, 7, 8]. The increased toxicity associated with centrally located tumors is the focus of the current prospective RTOG 0813 and EORTC 22113-0813 Lungtech trials, which were developed to identify a safe and effective dose-fractionation schedule for centrally located early stage NSCLC [9]. Although the high doses used for early stage lung cancer SBRT not surprisingly achieve high local control rates, the risk of toxicity is arguably unacceptable in the metastatic patient population [10]. The balance in the metastatic cohort must still shift in favor of toxicity minimization until prospective evidence supporting a survival benefit exists, which can warrant the increased side effect profile. This is especially true for those patients with high-risk central lung metastases where toxicity may be even more significant.

Many analyses of centrally located tumors treated with SBRT have defined “central” tumors as within 2 cm of the mainstem bronchus or other mediastinal structures [5–7, 11]. This is the most common definition utilized, which was initially defined by Timmerman et al. and is currently employed in RTOG 0813 [5]. Nevertheless, within this group exists an even more dangerous variant with actual tumor involvement of the mainstem bronchus. In fact, these tumors were deemed so dangerous that the EORTC 22113-0813 Lungtech trial excluded definitive SBRT of tumors with invasion of the proximal bronchial tree and/or hilar structures [9]. A recent 18 patient cohort analyzed by Haseltine et al. noted significantly higher SBRT-related toxicity and death in those patients with tumors abutting the proximal bronchial tree [12]. Alternatively, Stanford reported a smaller cohort of 7 patients with “ultra-central” tumors directly abutting the proximal bronchial tree or trachea and found no significant difference in 2-year overall survival, local control, or treatment-related toxicities relative to other central and peripheral lung lesions [13]. As systemic therapy options continue to improve there is a potential for longer-term survival in the metastatic patient population, which may be augmented further by the radiotherapeutic ablation of larger tumor sites [1, 14]. In this retrospective analysis we investigate the clinical outcomes of 20 patients diagnosed with advanced “high-risk” metastatic tumors with actual abutment or invasion of the mainstem bronchus treated with SBRT at Georgetown University Hospital.

## Materials and methods

### Patient eligibility

The MedStar Health Research Institute Georgetown University Oncology Institutional Review Board, approved this retrospective analysis of an established departmental treatment approach. Twenty consecutive patients with inoperable metastatic lung lesions localized to the central pulmonary tree were treated in 5 fractions with the SBRT CyberKnife radiosurgical system. The majority of metastatic lung lesions were confirmed pathologically prior to treatment when clinically prudent. Prudence was assessed by the interventional pulmonologist and included the accessibility of the lesion (including the safety of said biopsy), performance status of the patient, and/or lack of previous pathologic confirmation of metastatic disease. High-risk central lung lesions were defined as those abutting and/or invading the left or right mainstem bronchus [15]. All patients were evaluated by the same pulmonologist prior to treatment (EDA). Treatment with the CyberKnife SBRT system required the placement of 3 to 5 non-collinear fiducials within or directly adjacent to the tumor for tracking purposes [15, 16]. All patients underwent bronchoscopic placement of fiducials by the same pulmonologist (EDA). Patients with primary localized lung cancers or those with previous in-field thoracic irradiation were excluded from this study.

### Treatment planning and delivery

A fine-cut treatment planning CT scan was obtained in the supine treatment position for each patient using a GE LightSpeed RT16. CT scans were obtained with intravenous and/or oral contrast when clinically feasible and during full inhalation. Additional imaging including PET/CT scan was obtained as clinically indicated at the discretion of the treating physician (BCT). Gross tumor volumes (GTV) were contoured often with input from the attending pulmonologist. A 5 mm expansion from GTV to planning target volume (PTV) was added at the discretion of the attending radiation oncologist (BCT). A treatment plan was generated using the MultiPlan 5.2.1 non-isocentric inverse-planning algorithm. Radiation was delivered in 5 equal fractions of 7 or 8 Gy prescribed to an isodose line that covered at least 95 % of the PTV. Patients were treated in the supine position with their arms at their sides. Treatment was delivered over 5 consecutive days. Of note, treatment duration was calculated as the number of days from first to last treatment fraction.

The Synchrony Respiratory Motion Tracking System was utilized for both inter- and intrafraction tracking to accommodate for patient-specific respiratory motion. In this system, surface respiratory motion is monitored with external light-emitting markers attached to a patient-vest that is worn during treatment. These external markers are continuously monitored during treatment with a camera array and correlated with the internally placed fiducial markers tracked during treatment with orthogonal x-rays. In effect, an adaptive respiratory model is created during treatment for tumor position verification [15].

### Follow-up

Patients were followed with physical examination and CT+/−PET imaging at 3 to 6 month intervals per routine institutional practice. Palliative response was evaluated with a combination of radiation oncology, pulmonology, and medical oncology follow up documentation. Local tumor recurrence was defined as tumor progression evaluated on follow up radiological imaging. Local tumor progression was based on official radiological review and included increased tumor size, contrast enhancement, and/or mass effect. Overall survival and local control were measured from the date of treatment completion to the date of patient death and radiological progression or death, respectively. Toxicities were scored according to the National Cancer Institute Common Terminology Criteria for Adverse Events, Version 3.0 with acute and late toxicities separated at day 90.

### Statistical analysis

Statistical analysis was performed with the IBM SPSS Statistics version 22 (IBM Corporation and other(s) 1989, 2013). Dosimetric toxicity analysis was performed utilizing the Student’s *t*-Test (single-tailed, equal variance distribution). Actuarial local control and overall survival were calculated using the Kaplan-Meier method.

## Results

### Patient characteristics

Twenty patients with a median age of 66 years (range, 24 to 82 years) were treated from November 2008 to November 2011 at Georgetown University Hospital. The gender distribution was equal and the median pretreatment ECOG performance status was 0. Distribution of metastatic histologies included the following: 7 adenocarcinoma, 4 squamous cell carcinoma, 3 renal cell carcinoma, 2 carcinoid, 2 sarcoma (Ewing’s and leiomyosarcoma), 1 adenoid cystic, and 1 hepatocellular. The location of the primary tumors included the following: 7 pulmonary, 4 gastrointestinal, 3 head and neck, 3 pelvic, and 3 genitourinary. Isolated intrathoracic disease was noted in of 35 % of patients, and additional extrathoracic metastatic disease was present in the remaining 65 %. Pre-radiotherapy ipsilateral thoracic surgery and chemotherapy was noted in 60 and 75 % of patients, respectively. Specific patient characteristics are shown in Table 1.

**Table 1:** Patient characteristics

Characteristic	No. patients (%)
Age	
< 60	8 (40)
≥ 60	12 (60)
Gender	
Male	10 (50)
Female	10 (50)
ECOG performance status	
0	11 (55)
1	9 (45)
Histology	
Adenocarcinoma	7 (35)
Squamous	4 (20)
Renal cell	3 (15)
Sarcoma	2 (10)
Carcinoid	2 (10)
Other	2 (10)
Intrathoracic disease	
Singular	10 (50)
Multiple	10 (50)
Controlled extrathoracic disease	
Yes	13 (65)
No	7 (35)
Extrathoracic organ involvement	
Visceral	4 (20)
Bone	3 (15)
None	13 (65)
Lung location	
Right	13 (65)
Left	7 (35)
Pre-radiotherapy symptoms	
Shortness of breath	11 (55)
Cough	9 (45)
Hemoptysis	3 (15)
None	6 (30)

### Treatment characteristics

Patients were treated using the CyberKnife SBRT system to a median total dose of 40 Gy (35 or 40 Gy) all in 5 fractions, which corresponded to a biologic equivalent dose (BED_10_) of 59.5 and 72 Gy, respectively. Treatment was delivered to a median prescription isodose line of 75.5 % with median GTV target coverage of 98 % and average conformality index of 1.53. Mean tumor volume treated (PTV) was 111.3 cc (range, 22.6 to 300.0 cc) and median tumor volume treated was 85.8 cc. Treatment plans were composed of hundreds of pencil beams delivered using a single 20 to 40 mm diameter collimator. Median treatment duration from start to completion of SBRT was 7 days (range, 5 to 8 days). Mean maximum point doses delivered to the mainstem bronchus and esophagus were 46.7 and 28.7 Gy, respectively. Mean maximum point doses delivered to the spinal cord and left ventricle were 12.7 and 14.9 Gy, respectively. Mean and median total lung V15 Gy was measured to be 399.4 and 312.5 cc, respectively. Specific treatment and dosimetric characteristics are shown in Table 2.

**Table 2:** Treatment and dosimetric characteristics

Characteristic	No. patients (%)	Characteristic	No. patients (%)
Prior thoracic surgery		Prescription dose (Gy)	
Yes	12 (60)	35	8 (40)
No	8 (40)	40	12 (60)
Prior chemotherapy		Max point dose (Gy)	
Yes	15 (75)	Mean	48.3
No	5 (25)	Median	46.7
PTV volume (cc)		Range	41.7–57.1
Mean	111.3	Major bronchus max dose (Gy)	
Median	85.8	Mean	46.7
Range	22.6–300.0	Median	46.4
Conformality index		Range	40.0–50.0
Mean	1.53	Esophagus max dose (Gy)	
Median	1.50	Mean	28.7
Range	1.21–1.93	Median	31.1
Rx isodose line (%)		Range	10.8–40.6
Mean	75.5	Spinal cord max dose (Gy)	
Median	75.2	Mean	12.7
Range	70.0–80.0	Median	13.7
PTV coverage (%)		Range	3.2–19.3
Mean	98.0	Left ventricle max dose (Gy)	
Median	98.0	Mean	14.9
Range	96–100	Range	5.1–37.3
Treatment length	Days	Total lung V15 Gy (cc)	
Median	7	Mean	399.4
Range	5–8	Range	145.0–1005.0

### Palliative response

Fourteen patients had documented symptomatic central pulmonary metastases prior to treatment. The remaining patients were asymptomatic but were treated due to the compromising location and singular progression of the metastatic lesion. Pretreatment symptoms included shortness of breath (55 %), cough (45 %), and/or hemoptysis (15 %) as seen in Table 1. Nine patients (64 %) who were symptomatic prior to radiotherapy received moderate to significant symptomatic palliation after SBRT treatment. In the 5 symptomatic patients who received no palliation 2 developed late radiation toxicity, 1 developed progressive non-local symptomatic thoracic disease, and 1 failed locally.

### Treatment toxicity

Acute grade 2 or higher toxicity was noted in 1 patient who developed medically manageable esophagitis (grade 2). Documented late grade 2 or higher radiation toxicity was observed in 30 % of treated patients and included the following: grade 2 atelectasis (3), grade 2 bronchitis (1), grade 3 pneumonitis (1), and grade 4 atelectasis (1). Interestingly, all 5 late toxicities occurred in patients with right-sided tumors. Atelectasis was the most common late toxicity observed. One patient of the 5 who did not undergo pre-SBRT chemotherapy developed late grade 4 atelectasis. The remaining four grade 2 or higher late toxicities occurred in those patients who received pre-treatment chemotherapy (33 % of the chemotherapy cohort). Dosimetric univariate analysis of the cohorts who did and did not develop late atelectasis revealed prescription dose (*p* = 0.031), maximum point dose (51.9 vs. 47.4 Gy, *p* = 0.031), and major bronchus maximum point dose (49.0 vs. 46.1 Gy, *p* = 0.029) to be the only statistically significant predictors of late grade 2 or higher atelectasis. The one case of grade 4 atelectasis resulted in complete collapse of the ipsilateral lung requiring urgent bronchoscopic intervention. Specific late toxicity data is shown in Table 3.

**Table 3:** Specific late treatment toxicity

Histology	Primary location	Side	PTV (cc)	Rx dose (Gy)	Maximum point dose (Gy)	Major bronchus max dose (Gy)	Late toxicity
Squamous cell	Pulmonary	R	79.0	35	46.7	46.7	Grade 3 pneumonitis
Carcinoid	Pulmonary	R	95.0	35	46.1	46.1	Grade 2 atelectasis
Squamous cell	Head & neck	R	112	40	52.0	50.0	Grade 2 atelectasis
Adenocarcinoma	Uterine	R	63.0	40	52.6	50.0	Grade 2 atelectasis
Carcinoid	Pulmonary	R	126	40	57.1	50.0	Grade 4 atelectasis
Leiomyosarcoma	Uterine	R	162	35	46.7	46.0	Grade 2 bronchitis

### Local control and overall survival

**Fig. 1:**
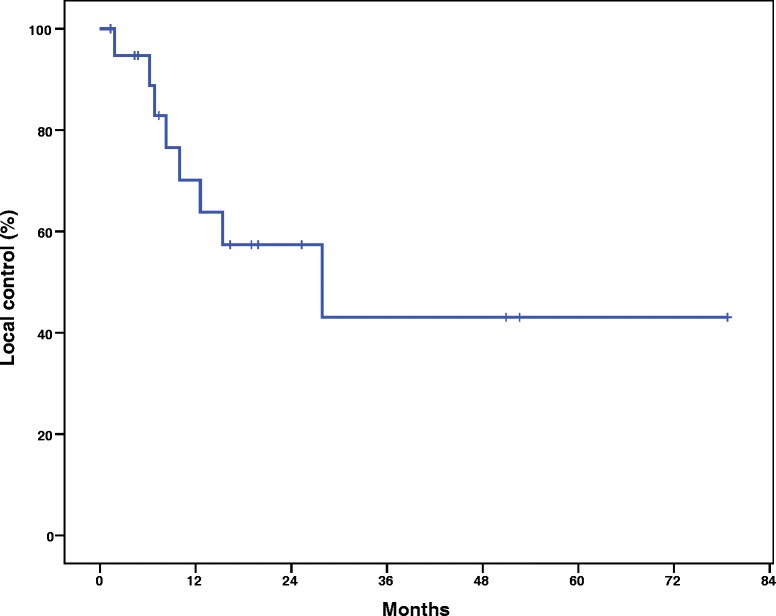
Kaplan-Meier local control from time of completion of SBRT

**Fig. 2:**
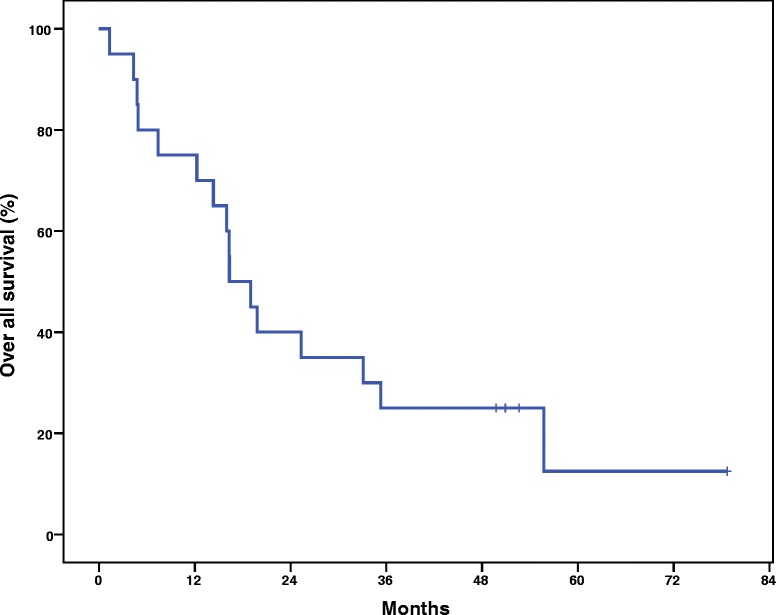
Kaplan-Meier overall survival from time of completion of SBRT

**Fig. 3:**
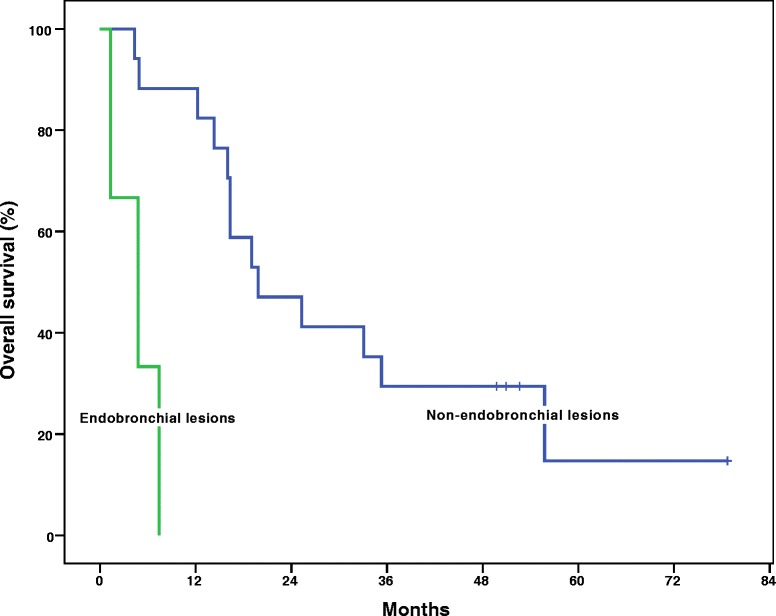
Kaplan-Meier overall survival from time of completion of SBRT of those patients with and without gross endobronchial involvement

**Fig. 4:**
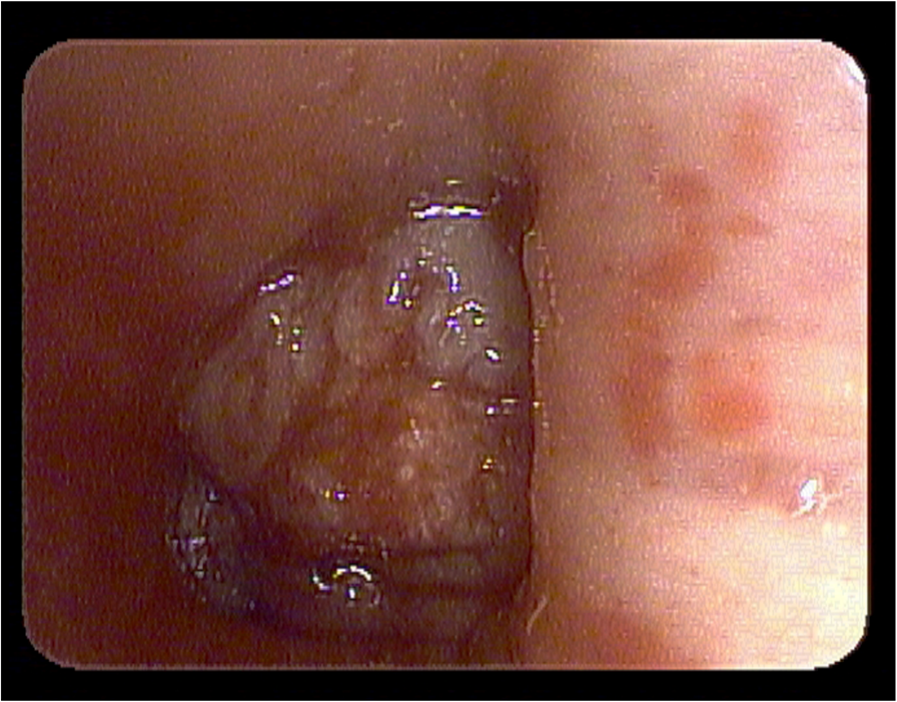
Bronchoscopic pre-radiotherapy image of a patient with gross endobronchial involvement of a central pulmonary metastasis

At a median follow up of 19 months, the 1- and 2-year Kaplan-Meier local control was 70.1 and 57.4 %, respectively (Fig. 1). The median Kaplan-Meier local control was estimated to be 27.9 months. The 1- and 2-year Kaplan-Meier overall survival was 75 and 40 %, respectively (Fig. 2). The median Kaplan-Meier overall survival was estimated to be 16.3 months. There was no significant difference in local control duration between 35 and 40 Gy fractionation schedules (17.2 vs. 20.7 months, *p* = 0.36) or size less or greater than median PTV of 85.8 cc (21.1 vs. 17.5 months, *p* = 0.35). However, there was a trend towards decreased local control in the GI versus non-GI primaries (8.5 vs. 22.0 months, *p* = 0.12). The majority of patients (11) died of progressive extrathoracic metastatic disease following radiotherapy treatment. Six patients died of progressive intrathoracic disease and 3 patients were still alive at the time of analysis. Of note, 3 patients were documented to have invasive endobronchial lesions prior to treatment and this very small cohort exhibited a significant decrease in median overall survival (4.8 vs. 19.8 months, *p* = 0.029), as seen in Fig. 3, and a trend in decreased local control (4.49 vs. 21.9 months, *p* = 0.085). Figure 4 illustrates a bronchoscopic view of one such invasive endobronchial lesion prior to SBRT.

## Discussion

Metastatic lesions of the central pulmonary tree negatively impact a patient’s quality of life and present complex radiobiological challenges for the treating radiation oncologist. Given their sensitive location these lesions can provoke severe cough, shortness of breath, and/or hemoptysis. Although chemotherapy is typically first-line treatment for metastatic disease it is unlikely to provide durable local control for such lung metastases [4]. Radiotherapy offers a very effective treatment option for both palliation and local control. SBRT has emerged as an ideal treatment modality given the excellent local control rates observed in the definitive treatment of early stage inoperable NSCLC [10]. Particularly important in the metastatic setting, SBRT offers a shorter treatment length yielding a minimal break if any of systemic therapy, which is still the backbone of treatment for the metastatic patient. SBRT for lung metastases has been previously shown to yield effective local control with minimal toxicity [17]. Nevertheless, Timmerman et al. reported higher rates of treatment-related toxicity in central relative to peripheral primary NSCLC tumors in the definitive setting [5]. Moreover, Baumann et al. demonstrated inferior rates of local control in central relative to peripheral tumors [18]. Conversely, recent accumulating retrospective data of admittedly heterogeneous dose and fractionation schedules seems to indicate the danger of irradiation to this central region may not have as clinically significant an impact as previously postulated [13, 19–21]. Broadly speaking treatment-related toxicities can be seen in several anatomic locations including: mainstem bronchus, lung parenchyma, esophagus, brachial plexus and vagus nerve, and chest wall [8]. Many of these toxicities can be limited by placing dosimetric constraints on adjacent organs, however central tumors are often intricately involved with mainstem bronchi making achieving these constraints more difficult.

A European analysis of factors contributing to the efficacy of SBRT for inoperable stage I NSCLC found that central lung tumors were both more likely to fail locally and to develop atelectasis as a treatment-related toxicity [18]. Likewise, the most common late toxicity observed in our patient population was atelectasis. Interestingly, Joyner et al. reported major airway wall thickening evident on follow up CT scans as a common observation in their cohort of central lung tumors treated with SBRT [11]. Although the pathophysiology of this atelectasis is not firmly established in the literature, it is hypothesized to be secondary to dose-dependent radiation-induced damage of the bronchial wall [8]. This dose-dependence is consistent with our observation of maximum point dose to the major bronchus as the most significant predictor of late grade 2 or higher atelectasis. Given the hypothesized etiology of bronchial wall damage it may be prudent to consider the major bronchus as a serial organ at risk and make concerted efforts to avoid hot spot placement in this region. As toxicity and radiological data accumulates an evolution in our understanding of the bronchial wall as a dose limiting structure is paramount.

It is important to note that not all central pulmonary lesions are created equally. Hamamoto et al. demonstrated that metastatic lung lesions rather than NSCLC primary lesions tend to have higher rates of local recurrence [22]. Additionally, Milano et al. reported lower rates of local control in pulmonary metastases of gastrointestinal origin [23]. Similarly, Singh et al. reported outcomes of 5-fraction SBRT to a higher total dose (50 Gy) for treatment of oligometastatic thoracic disease and found all 5 patients with local failures were those with colorectal primaries [24]. Although, in the present study there was no significant difference in duration of local control or overall survival between metastases of GI and non-GI origin (*p* = 0.12 and *p* = 0.23, respectively), there was a trend towards increased local progression in the GI subset. Consequently, it is crucial to note that tumors with lower α/β may necessitate altered fractionation schemes to achieve durable local control.

In a similar study, Oshiro et al. reported their single institutional experience in a retrospective review of 21 patients who all received linac-based SBRT to the pulmonary hilum [7]. In this study, both metastatic and primary hilar lung tumors were treated to a median dose of 50 Gy in 5 fractions (range of 25 to 60 Gy in 1 to 13 fractions). Hilar metastases in this study were defined as within 2 cm of the mainstem bronchus. Reported local control rates of 74.3 and 59.6 % were observed at 1 and 2 years, respectively. There were no reported grade 2 or above acute toxicities. Observed late grade 3 or above toxicities included 1 chronic dyspnea requiring supplemental oxygen, 1 recurrent bronchial obstruction causing intractable cough, and 1 fatal hemoptysis. Of note, the one reported fatal hemoptysis occurred in a patient who was previously treated twice with thoracic radiation. Notwithstanding the fact that tumors in our study were intricately involved with the central airway rather than just within 2 cm, reported grade 3 or higher late toxicities were less frequent. Additionally, the 12-month local control rate of 74.3 % is similar to that reported in our study (70.1 %) despite the lower median dose delivered to our cohort.

Our 2-year local control rate of 57.4 % is notably inferior when compared broadly with that seen in SBRT-treated central tumors elsewhere in the literature (64 to 95 %) [21]. This is likely a reflection of the following factors: (1) SBRT treatment of metastatic pulmonary lesions demonstrates inferior local control relative to primary NSCLC, (2) the high-risk nature of these tumors in comparison to standard-risk or RTOG defined central tumors, and (3) the lower BED employed in their treatment [9, 21, 22, 25]. Total BED may be the root cause of this inferior local control. A very large (613 patient) retrospective German analysis of centrally located stage I NSCLC found significantly lower BEDs were used to treat central versus peripheral tumors (72.0 versus 84.4 Gy, respectively) and this resulted in a notably lower 3-year freedom from local progression rate (52 versus 84 %, respectively) [25]. There is an indication from a small 7 patient Stanford cohort of “ultra-central” tumors that these high-risk lesions may be successfully treated with higher doses (median 12.5 Gy × 4) to achieve excellent 2-year local control of 100 % without significant increase in toxicity [13]. Of note, 6 of the 7 ultra-central tumors were of primary rather than metastatic histology and would be expected to have higher local control rates compared to metastatic lesions. Overall, data regarding the safety and efficacy of SBRT to high-risk central tumors is scant, and additional research is essential to determine if dose escalation is safe and can achieve superior local control.

As our understanding of the optimal SBRT treatment for central pulmonary lesions evolves, recent data suggests treatment of these lesions may not be as dangerous as previously suspected. Mangona et al. reported the results of a propensity score matched-pair analysis of 158 central and peripheral lung tumors (primary or metastatic) treated with SBRT (range of 48 to 60 Gy in 4 to 5 fractions) [19]. Central tumors were defined as within 2 cm of the proximal bronchial tree or PTV touching the mediastinum. Interestingly, there was no observed significant difference in any 2-year reported adverse event based on tumor location. Moreover, the 2-year incidence of grade 4 and 5 adverse events was exceedingly low at <1 and 0 %, respectively. Park et al. retrospectively investigated a cohort of 251 patients with similarly defined central NSCLC primary tumors treated with SBRT [20]. Although patients in the central lesion cohort had significantly larger tumors and were of older age, multivariate analysis found central tumor location was not associated with inferior toxicity, local control, or overall survival. Finally, Davis et al. published a 111 patient cohort with central primary or metastatic lung tumors treated with SBRT and found no grade 3 or higher acute or late toxicities [21]. Nevertheless, they did report a lower 2-year rate of local control in metastatic versus primary lung lesions (69.8 versus 76.4 %) as would be expected. Additional prospective data with appreciable follow-up will be necessary to confirm if these intriguing modern retrospective reports of minimal SBRT-related toxicity are reproducible.

Endobronchial lesions may represent the highest of high-risk pulmonary metastatic lesions. Survival after diagnosis of endobronchial tumors is generally poor and in our admittedly very small cohort these lesions were shown to have significantly lower overall survival relative to non-endobronchial high-risk central lesions [26]. Overall, diagnosis of endobronchial metastatic tumors is quite rare with autopsy series reporting a 2 % incidence in solid malignancies [27]. These lesions are most commonly secondary to lung cancers, but can also arise from metastatic breast, colon, or renal cell carcinoma [26–28]. Much of the literature surrounding management and clinical outcomes of endobronchial lesions is limited to case reports and retrospective reviews of small cohorts. There is some data to suggest these lesions respond poorly to systemic therapy and can lead to meager median survivals as low as 9 months [29]. Nevertheless, these lesions tend to be quite symptomatic prior to treatment and significant mitigation of symptoms can be achieved with local therapy [29].

Our study reports the efficacy of a 5-fraction SBRT treatment regimen for high-risk central pulmonary metastases. Of the patients who had symptomatic lesions prior to treatment the majority (64 %) had effective mitigation of their symptoms. Relatively durable tumor control was observed with a 12-month local control of 70.1 %, which translated into a 1-year overall survival of 75 %. Late grade 2 or higher atelectasis was the most common treatment-related toxicity and correlated significantly with a increased maximum point dose to the major bronchus. Furthermore, those patients with invasive endobronchial lesions demonstrated a significantly lower overall survival and a trend towards shorter local control. Limitations of the present study include the small patient population, the heterogeneity of metastatic histologies, and its retrospective analysis. As evidence accumulates regarding the lower than expected toxicity of SBRT treatment of central lung tumors the investigation of dose escalation for these high-risk lesions seems warranted in attempt to achieve superior local control closer to that seen in standard-risk central lung tumors. Reports from the RTOG 0813 and EORTC 22113-0813 Lungtech trials exploring the proper SBRT fractionation schedule for central primary lung tumors may be extrapolated to help address the optimal fractionation schedule for precarious central pulmonary metastatic lesions.

## Conclusion

Five-fraction SBRT to a total dose of 35 or 40 Gy appears to be a safe and effective management strategy for high-risk metastatic lesions of the central pulmonary tree. Care should be taken to limit the maximum point dose to the mainstem bronchus to avoid late radiation-related toxicity. The role of radiotherapy in the treatment of these precarious central pulmonary lesions requires further prospective exploration to establish the efficacy, safety, and proper fractionation schedule.
